# CaMKII as a Therapeutic Target in Cardiovascular Disease

**DOI:** 10.1146/annurev-pharmtox-051421-111814

**Published:** 2022-08-16

**Authors:** Oscar E. Reyes Gaido, Lubika J. Nkashama, Kate L. Schole, Qinchuan Wang, Priya Umapathi, Olurotimi O. Mesubi, Klitos Konstantinidis, Elizabeth D. Luczak, Mark E. Anderson

**Affiliations:** 1 Department of Medicine, The Johns Hopkins University School of Medicine, Baltimore, Maryland, USA; 2 Cleveland Clinic Lerner College of Medicine, Cleveland, Ohio, USA; 3 Departments of Physiology and Genetic Medicine and Program in Cellular and Molecular Medicine, The Johns Hopkins University School of Medicine, Baltimore, Maryland, USA

**Keywords:** CaMKII, cardiovascular disease, kinase, calcium, heart failure, arrhythmias

## Abstract

CaMKII (the multifunctional Ca^2+^ and calmodulin-dependent protein kinase II) is a highly validated signal for promoting a variety of common diseases, particularly in the cardiovascular system. Despite substantial amounts of convincing preclinical data, CaMKII inhibitors have yet to emerge in clinical practice. Therapeutic inhibition is challenged by the diversity of CaMKII isoforms and splice variants and by physiological CaMKII activity that contributes to learning and memory. Thus, uncoupling the harmful and beneficial aspects of CaMKII will be paramount to developing effective therapies. In the last decade, several targeting strategies have emerged, including small molecules, peptides, and nucleotides, which hold promise in discriminating pathological from physiological CaMKII activity. Here we review the cellular and molecular biology of CaMKII, discuss its role in physiological and pathological signaling, and consider new findings and approaches for developing CaMKII therapeutics.

## INTRODUCTION

The multifunctional Ca^2+^ and calmodulin-dependent protein kinase II (CaMKII) was discovered nearly half a century ago (1). While much is now understood about its molecular physiology and role in health and disease, major questions remain. The diverse nature of the target proteins gives CaMKII broad regulatory input into fundamental cellular processes. The multifunctional nature of these roles is determined by the cellular and subcellular sites of CaMKII activity. In mammals, CaMKII exists as four distinct isoforms and multiple splice variants that are heterogeneously distributed in cells and organs. In addition, CaMKII activity is tuned, directly and indirectly, and contributes to both activator and inhibitor signals. The consequent richness of CaMKII signaling thus helps to precisely orchestrate a broad range of biological activities. CaMKII was initially celebrated for its role in learning and memory but is now recognized to support a range of processes related to fight-or-flight physiology. At the same time, CaMKII is one of the most preclinically validated signals for triggering cardiovascular and other diseases. The potential for CaMKII to become a therapeutic target has heightened the stakes for understanding the roles of CaMKII in health and disease and for developing improved proof-of-concept therapies to better understand the opportunities and pitfalls of CaMKII inhibition. Here we highlight structure-activity relationships of CaMKII and its regulatory posttranslational modifications (PTMs), the role of CaMKII in the cardiovascular system, how CaMKII evolved reactive oxygen sensing that enhances physiological performance while simultaneously augmenting the potential for CaMKII to promote disease, and considerations for CaMKII as a therapeutic target.

## CAMKII STRUCTURE AND FUNCTION

CaMKII is a central integrator of upstream cellular signals, including Ca^2+^ influx, oxidative and nitrosative stress, and hyperglycemia. By phosphorylating various downstream targets, CaMKII translates these signals into physiological and pathological cellular responses by modulating intracellular Ca^2+^ dynamics, contractility, metabolism, and gene expression.

**Figure 1:**
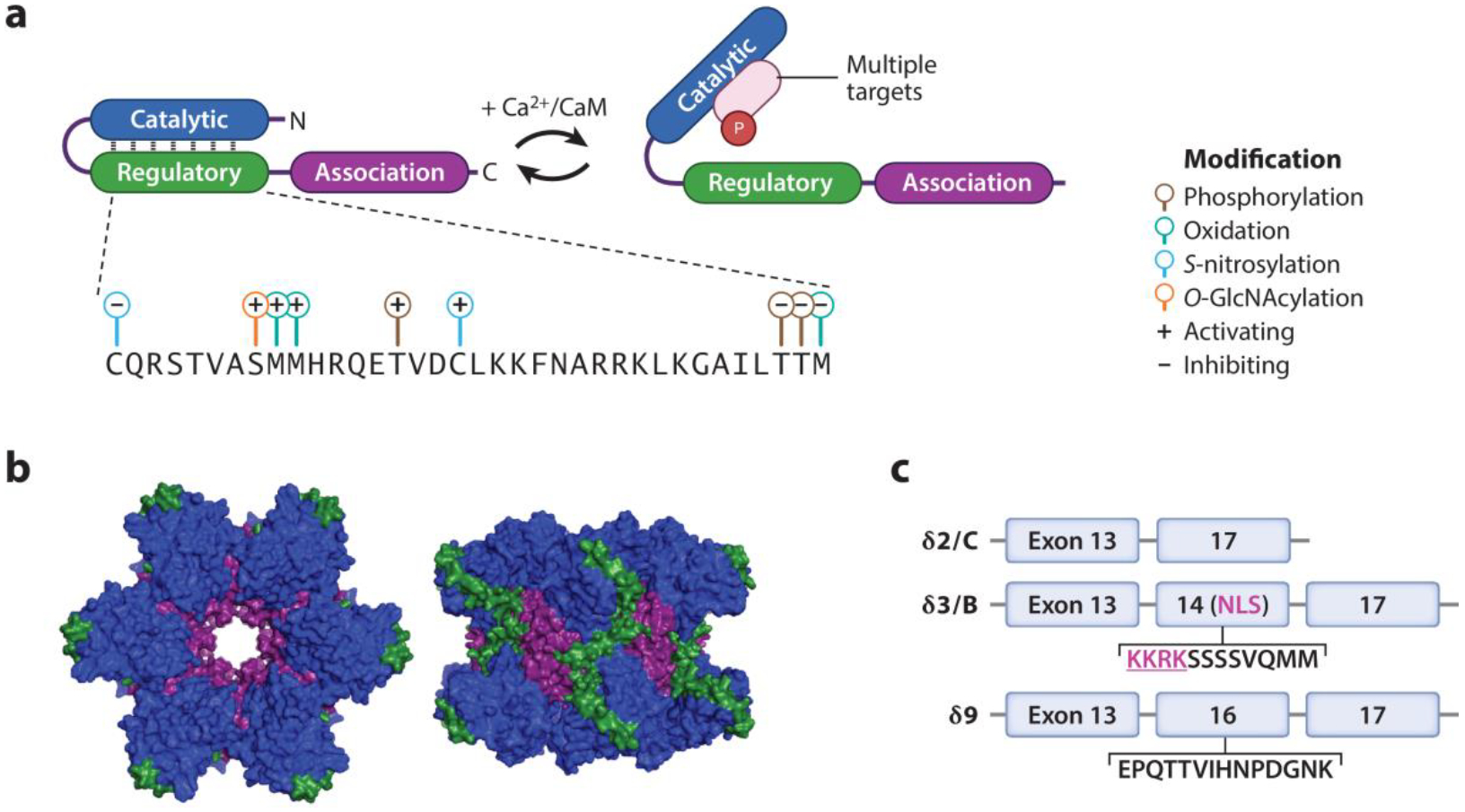
Multifunctional Ca^2+^ and calmodulin-dependent protein kinase II (CaMKII) structure and function. (*a*) The schematic of a CaMKII monomer shows that, upon binding Ca^2+^/calmodulin (CaM), the catalytic domain is released from the autoinhibitory pseudosubstrate and is enabled to phosphorylate its downstream targets. Several posttranslational modifications in the regulatory domain positively and negatively modulate CaMKII activity. Panel adapted from image created with Biorender.com. (*b*) In this structural depiction of CaMKII dodecameric holoenzymes, each domain is color coded as in panel *a*. Structures from the RCSB Protein Data Bank, rcsb.org (PDB IDs 5VLO, 2VN9, and 3SOA) (CC0 1.0). (*c*) CaMKIIδ undergoes alternative splicing to generate three prevalent variants in the heart. These variants differ only at the variable linker by inclusion or omission of exons 14 or 16.

In mammals, CaMKII is present in four isoforms (α, β, δ, γ), each encoded by their own gene. While highly homologous, the isoforms exhibit distinct tissue distribution and physiological functions (2). Myocardium primarily expresses CaMKIIδ, with minor contribution from CaMKIIγ, whereas CaMKIIα and CaMKIIβ are mainly expressed in the central nervous system (3, 4). Each CaMKII molecule is composed of an N-terminal catalytic domain, a central regulatory domain, and a C-terminal association domain (Figure 1a). The catalytic domain contains the substrate and ATP binding pockets. The regulatory domain contains the autoinhibitory pseudosubstrate, a calmodulin (CaM) binding motif, and several sites for PTMs. Lastly, the association domain allows for CaMKII to assemble as a dodecameric holoenzyme (Figure 1b).

Initial activation occurs when intracellular Ca^2+^ increases, leading to calcified CaM (Ca^2+^/CaM) binding to CaMKII and displacement of the pseudosubstrate from the catalytic domain granting it access to substrates and ATP. Transient activation is reversible: A decrease in Ca^2+^ (as occurs in myocardium during each diastolic interval) allows CaM to decalcify and unbind from CaMKII, whereupon the pseudosubstrate reinhibits kinase activity. In contrast, persistent activation due to sustained intracellular Ca^2+^ elevation [as occurs during rapid heart rates (tachycardia) or action potential prolongation in myocardium] allows CaMKII to autophosphorylate at threonine 287 (T286 in CaMKIIα) (5–7) (Figure 1a). T287 autophosphorylation renders CaMKII autonomous from regulation by Ca^2+^ by two mechanisms: It causes CaM trapping by increasing affinity to CaM by a thousandfold, and it decreases the affinity of the pseudosubstrate for the catalytic domain (8). Autophosphorylation can spread throughout a holoenzyme, depending on the activity of countervailing phosphatases, since an active catalytic domain phosphorylates neighboring CaMKII monomers within the holoenzyme (9, 10).

## CAMKII REGULATION

### Upstream Regulators and Posttranslational Modifications

Several upstream regulators, including catecholamines, angiotensin II, aldosterone, nitric oxide, and hyperglycemia, are known to modulate CaMKII activity via activating and inhibiting PTMs (Figure 1a). β-Adrenergic stimulation leads to enhanced intracellular Ca^2+^ and nitric oxide production, which increase CaMKII activity via T287 autophosphorylation and C290 nitrosylation, respectively (11–14). In contrast, CaMKII autophosphorylation at T306/T307 has been shown to reduce Ca^2+^/CaM binding (15, 16). Angiotensin II (17–19) and aldosterone (20) drive CaMKII activation by inducing reactive oxygen species (ROS) generation via nicotinamide adenine dinucleotide phosphate oxidases and mitochondria. Oxidative activation of CaMKII (ox-CaMKII) occurs at M281/282 and can be reversed by cellular methionine sulfoxide reductases (21). In contrast, inhibitory M308 oxidation prevents CaM trapping (21). Dynamic regulation of the redox state at M308 by molecule interacting with CasL 1 (MICAL1), a methionine monooxygenase, and methionine sulfoxide reductase B (MsRB) was recently shown by our group to tune CaMKII activity by controlling the avidity of Ca^2+^/CaM binding (22). In addition to being a ROS sensor, CaMKII also promotes ROS production (23–25), consistent with a potential role for ox-CaMKII in a ROS-induced positive feedback loop. Hyperglycemia has been shown to enhance *O*-linked-*N*-acetylglucosaminylation (*O*-GlcNAcylation) of CaMKII, and studies from the Bers group (18, 23, 26) suggest that this modification enhances CaMKII activity and downstream consequences such as spontaneous sarcoplasmic reticulum (SR) Ca^2+^ leak. In contrast, a study from our group found that loss of the *O*-GlcNAcylation site on CaMKII (S280) was insufficient to prevent arrhythmia in hyperglycemic mouse models (27). However, given the proximity of both the *O*-GlcNAcylation and oxidation sites on CaMKII, these modifications could be influencing each other, affecting the downstream consequences from either modification. The diversity of activating and inhibiting PTMs to CaMKII may offer an opportunity to modulate them therapeutically (see the section titled Indirect Targets).

### Cellular Distribution

In the heart, CaMKIIδ undergoes alternative splicing (28) (Figure 1c). While 11 splice variants (CaMKIIδ1–11) have been identified, CaMKIIδ2/C, CaMKIIδ3/B, and CaMKIIδ9 are the most abundant and best understood (29). These variants differ only by their linker region localized between the regulatory and association domains. CaMKIIδ3/B is primarily nuclear and contains a nuclear localization signal. In contrast, the other splice variants (CaMKIIδ2/C and CaMKIIδ9) are predominantly cytoplasmic and lack a nuclear localization signal. Intriguingly, CaMKII holoenzymes may be assembled as heteromultimers, containing subunits from different isoforms and splice variants. This heterogeneity can shift holoenzyme localization based on the predominant isoform. For example, when the holoenzyme is predominantly made of CaMKIIδ3/B, it will localize to the nucleus (30, 31). In myocardial cytosol, CaMKII has been observed to concentrate in discrete nanodomains in T-tubules and in close association with SR. Recently, Carlson et al. (32) found that AKAP18δ [redefined as CaM-kinase anchoring protein (CaM-KAP)] recruits CaMKII to the SR. CaMKII has also been detected in the matrix of mitochondria, where it plays a role in regulating mitochondrial bioenergetics and Ca^2+^ entry (33–35), although this latter function has been disputed (36, 37). While it has been suggested that the mitochondrial isoform is CaMKIIδ2/C, the identity of mitochondrial CaMKII remains unclear (33, 38, 39).

### Downstream Targets

**Figure 2:**
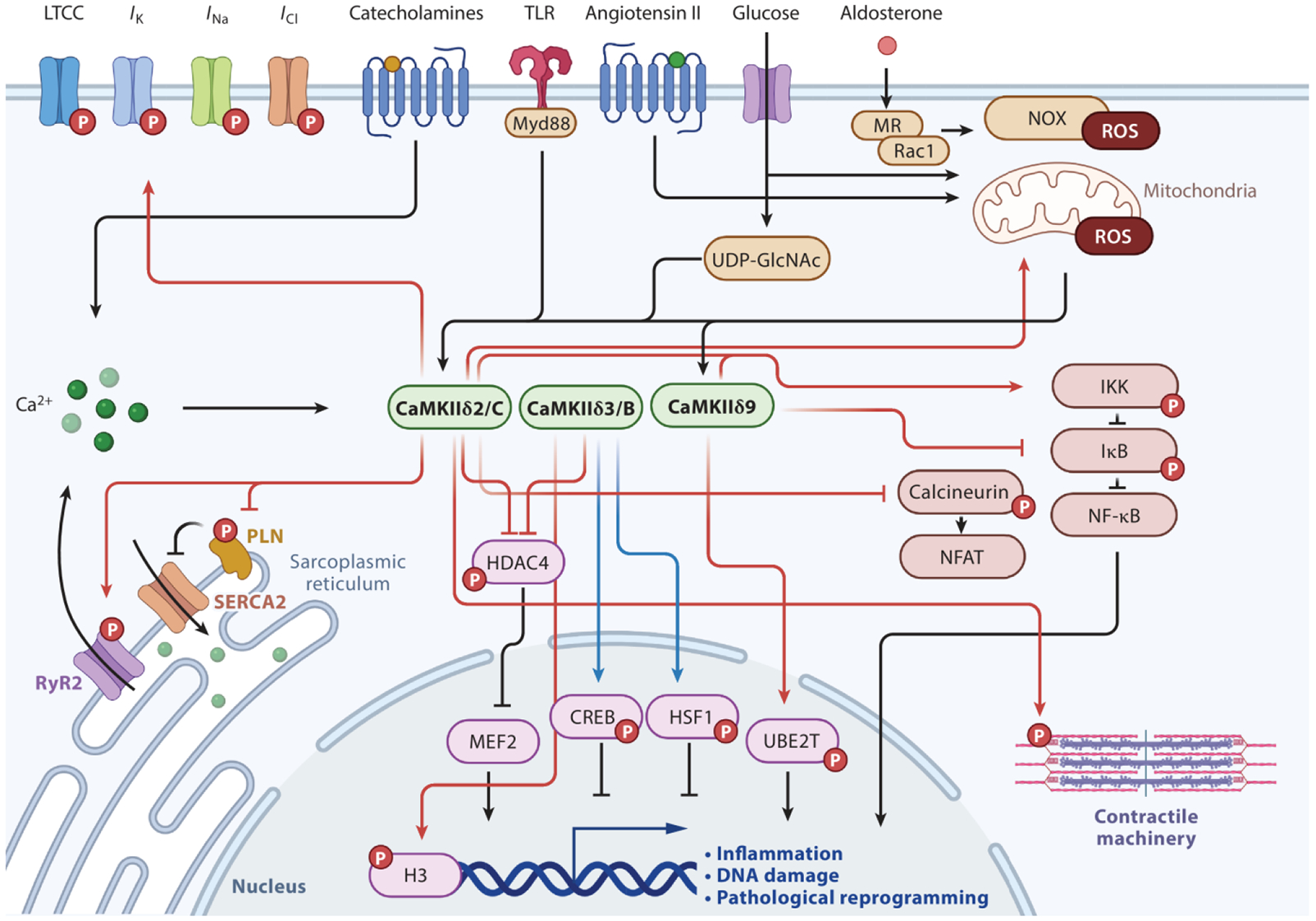
Major known CaMKII signaling pathways in cardiomyocytes. Black arrows denote upstream stimulants, and faded arrows denote downstream pathological (*red*) and cardioprotective (*blue*) targets. Red phosphate groups denote phosphorylation by CaMKII. When known, arrow origin denotes the responsible splice variant. Abbreviations: CaMKII, multifunctional Ca^2+^ and calmodulin-dependent protein kinase II; CREB, cAMP response element–binding protein; H3, histone 3; HDAC4, histone deacetylase 4; HSF1, heat shock factor 1; *I*, current; IκB, inhibitor of nuclear factor kappa-B; IKK, inhibitor of nuclear factor kappa-B kinase; LTCC, L-type calcium channel; MEF2, myocyte-enhancer factor 2; MR, mineralocorticoid receptor; Myd88, myeloid differentiation primary response 88; NFAT, nuclear factor of activated T cells; NF-κB, nuclear factor kappa-B; NOX, nicotinamide adenine dinucleotide phosphate oxidase; PLN, phospholamban; Rac1, Rac family small GTPase 1; ROS, reactive oxygen species; RyR2, type 2 ryanodine receptor; SERCA2, sarco/endoplasmic reticulum calcium ATPase 2; TLR, toll-like receptor; UBE2T, ubiquitin-conjugating enzyme E2 T; UDP-GlcNAc, uridine diphosphate *N*-acetylglucosamine. Figure adapted from image created with Biorender.com.

In states of increased physiological demand, CaMKII can phosphorylate and regulate many downstream pathways that influence intracellular Ca^2+^ concentration homeostasis, excitation-contraction coupling, gene expression, inflammation, metabolism, and cell death (Figure 2). Excessive and chronic CaMKII activity can turn acute adaptive signals into drivers of disease. CaMKII isoforms are known to have different targets and effects on cardiac pathophysiology. Mishra et al. (30) showed that the variants δ3/B and δ2/C can be differentially activated by upstream stimuli and can preferentially phosphorylate certain targets. Under the influence of neurohormonal agonists, CaMKII phosphorylates transcriptional modulators that promote pathologic gene programs—for example, histone deacetylase 4 (HDAC4) (40), histone 3 (41, 42), and nuclear factor κB (43–47)—or mediate cardioprotection, such as cAMP response element–binding protein (CREB) (48, 49) and heat shock factor 1 (HSF1) (50). In addition, CaMKII contributes to heart failure by interacting with the Fanconi anemia DNA repair pathway, leading to increased myocardial death. Zhang et al. (29) recently found that the CaMKδ9 variant impairs DNA repair by phosphorylating and promoting degradation of ubiquitin-conjugating enzyme E2 T (UBE2T). Thus, through multiple pathways, CaMKII regulates genetic stability and expression during injury.

CaMKII exerts its proarrhythmic signal through a broad number of ion channels and SR-associated proteins. CaMKII is known to activate L-type calcium channels (51, 52), various K^+^ channels (53–55), and cardiac voltage-gated sodium channel (Na_V_)1.5 (56) and Na_V_1.8 (57). Stimulation of these channels results in increased early and delayed afterdepolarizations and spatially dispersed repolarization that promotes cardiac arrhythmias such as atrial fibrillation and ventricular tachycardia and fibrillation. CaMKII phosphorylates the type 2 ryanodine receptor (RyR2) on serine S2814, promoting release of Ca^2+^ from the SR into the cytoplasm (58, 59). This SR Ca^2+^ leak can activate proarrhythmic Ca^2+^-sensitive conductances, most notably depolarizing the inward current carried by the sarcolemmal Na^+^/Ca^2+^ inward current (60). CaMKII also phosphorylates phospholamban (PLN) at Thr17, leading to its disengagement from sarco/endoplasmic reticulum calcium ATPase 2a (SERCA2a) and loss of inhibitory action (61). The actions of CaMKII at RyR2 and PLN appear to be coordinated by the binding protein AKAP18δ (32). Furthermore, unbiased mass spectrometry has identified upwards of 30 phosphoresidues downstream of isoproterenol-stimulated CaMKII in the heart (62). These targets included sarcomere proteins, kinases, transcription factors, and cytoskeletal regulators. However, whether these are bona fide or indirect targets remains to be determined.

### Activity Measurement

Methods to measure CaMKII activity are instrumental to elucidate its role in physiology and disease. Classically, CaMKII activity can be determined in solution by measuring the incorporation of radiolabeled ATP into a validated CaMKII substrate, such as Syntide-2 (63), or using luciferase-based assays that measure ATP consumption. Additionally, immunoblotting for CaMKII autophosphorylation at T287 (64) is commonly used. However, these techniques have many drawbacks, and several of the commercial antibodies for detecting T287 autophosphorylation are unreliable (65). Moreover, these are terminal assays that require tissue disruption, lack temporal and spatial resolution, and may introduce artifactual loss or incorporation of PTMs. In vivo biosensors address these issues and offer real-time tracking of CaMKII activity. Camui, the first CaMKII biosensor developed, is composed of a CaMKIIα monomer flanked by Förster resonance energy transfer (FRET) reporters (66, 67). Here, activation of CaMKII and subsequent dissociation of the catalytic from the regulatory domain cause a measurable decrease in FRET signal. Camui has been further optimized using improved fluorophores to yield a dynamic range of ~80% (68). Nevertheless, use of Camui has important caveats: It causes an inherent overexpression of CaMKIIα and does not report on bona fide activity; rather, it reports on conformational changes due to Ca^2+^/CaM binding to Camui as a proxy for CaMKII activity. The more recent biosensors FRESCA and CaMKII-KTR are substrate sensors, which circumvent some of these shortcomings (27, 69, 70). However, low sensitivity and dependence on nucleocytoplasmic transport to function (KTR) limit their utility. In summary, there remains a pressing need for high-performance CaMKII biosensors that can function both in vivo and in vitro.

## EVOLUTION, AGING, AND ANTAGONISTIC PLEIOTROPY

The evolutionary history of CaMKII can provide valuable insights with therapeutic relevance. PTM-modulated residues appear to have been added to CaMKII at different time points during evolution. We recently noted that the S280, responsible for *O*-GlcNAcylation-induced activation (18), is present in the Choanozoa and absent in the Filasterea, suggesting that it emerged after the divergence between these two lineages. Strikingly, we also found that the ROS-sensing residues C280/M281 (70) or M281/M282 (CM/MM) evolved in ancestral vertebrates about 650 million years after the emergence of CaMKII itself. The CM/MM residues are absent in nonvertebrate lineages but completely conserved throughout the vertebrate lineages. Intriguingly, knock-in insertion of the MM module to invertebrate CaMKII in *Drosophila melanogaster* confers the invertebrate CaMKII with the ability to transduce ROS signals in vivo (70). These results suggest that the cellular context for ROS-induced CaMKII activation was antecedent to vertebrates, preceding the evolution of the MM module. This vertebrate-specific role of CaMKII oxidation contrasts with T287 autophosphorylation, which is conserved from the unicellular Filasterea to humans. Conceivably, T287 autophosphorylation is involved in a wider range of physiological functions than oxidation of the CM/MM residues. Therefore, targeting CaMKII oxidation may represent a safer strategy than targeting CaMKII autophosphorylation or indiscriminate inhibition of CaMKII for specific conditions. This notion is further bolstered by our finding that the acquisition of ROS-sensing capability by the vertebrate CaMKII through the MM module is an example of antagonistic pleiotropy, an evolutionary theorem that predicts conservation of traits that provide early-life benefit despite later-life deleterious consequences. Specifically, we found that CaMKII oxidation enhances youthful fitness (such as increased muscle function) while promoting age-related diseases, frailty, and premature death during aging in both mice and MM module–containing flies (70). The late-onset detrimental effects of CaMKII oxidation support the concept that the therapeutic value of targeting ox-CaMKII may increase as we age. Targeting CaMKII oxidation may not only be effective against age-related diseases but potentially counter aging itself.

Another vertebrate-specific feature of CaMKII is that vertebrates have obtained at least four separate CaMKII genes through gene duplications (71). The presence of multiple CaMKII genes with nonidentical sequences and functions presents the opportunity for isoform-specific targeting. Such isoform-specific targeting might be useful when a specific CaMKII isoform plays a major role in the disease of interest. It might be achieved by isoform-selective drugs, gene therapies, and delivery of the therapeutic agents to selected tissues where the specific CaMKII gene is expressed (see the section titled CaMKII Antagonist Tools and Candidate Therapies). However, the redundant functions among the CaMKII isoforms also present challenges to such isoform-specific targeting because one CaMKII isoform may compensate for the inhibition of another, as was recently shown in zebrafish (72).

## CAMKII IN PHYSIOLOGY

### Long-Term Potentiation

One of the most well-studied physiological roles of CaMKII is its crucial function in long-term potentiation (LTP). LTP describes brief pulses of synaptic activity in neuronal cells that produce long-lasting changes in synaptic plasticity. CaMKII becomes active in neuronal spines following synaptic signaling by glutamate (73). CaMKII is recruited to the postsynaptic density via its interaction with *N*-methyl-d-aspartate-type glutamate receptor (74) and phosphorylates targets that trap freely diffusing AMPA receptors, a phenomenon that is also required for LTP (75, 76). The indispensable nature of CaMKII in LTP induction is underscored by the fact that mice with mutations in CaMKIIα experience learning and memory deficits (77–79). In addition to LTP induction, CaMKII has also been implicated in LTP maintenance and long-term depression (LTD), the opposing process to LTP that weakens synaptic strength. These various neuronal functions of CaMKII rely on different PTM phosphorylation sites on the kinase itself. More specifically, it has been shown that CaMKII phosphorylation at T286 in CaMKIIα is necessary for LTP induction but not maintenance (80–82). In contrast, LTD (stimulated by low-frequency stimulation of neurons) leads to T305/306 phosphorylation (83). Taken together, these studies demonstrate the important nature of CaMKII activity specification by PTMs on various processes underlying changes in synaptic plasticity.

### Pacemaking

CaMKII is present in sinoatrial node cardiac pacemaking cells and is localized to the SR upon activation (84). While early studies suggested that CaMKII activity is required for modulating the generation of action potentials in the sinoatrial node, it was more recently demonstrated that the role of CaMKII in pacemaking activity may be more specific to fight-or-flight physiology (84–86). In a transgenic mouse model expressing a CaMKII peptide inhibitor, autocamtide-3 derived inhibitory peptide (AC3-I), heart rates were similar to those of control mice under resting conditions but slower following β-adrenergic receptor stimulation by isoproterenol. Although CaMKII plays a facilitating role in pacemaker physiology, excessive ox-CaMKII can contribute to sinoatrial node pathology, mostly a disease of elderly patients, and serve as a possible example of antagonistic pleiotropy. Patients with sinoatrial node dysfunction and heart failure have increased levels of ox-CaMKII compared to those with heart failure alone (19). The role of CaMKII oxidation in pacing is complex, as exemplified by the recent discovery that MICAL1-catalyzed oxidation of M308 reduces heart rate responses associated with spontaneous activity and in response to isoproterenol (22). In addition, expression of AC3-I or CaMKIIN, an endogenous peptide inhibitor of CaMKII (87) discussed further below, in a transgenic mouse model provided protection against the reduction in resting heart rate and sinoatrial nodal cell damage resulting from angiotensin II infusion (19).

### Blood Pressure and Vascular Remodeling

CaMKII has been found to play several important regulatory roles in vascular biology, including the modulation of blood pressure, vascular tone, and vascular remodeling following injury. CaMKII was shown to influence angiotensin II–mediated hypertension, and its actions were abrogated in transgenic mice with vascular smooth muscle cell–targeted CaMKIIN overexpression (88). Interestingly, CaMKII helps maintain intracellular Ca^2+^ homeostasis in vascular smooth muscle cells but is not required for agonist-induced vasoconstriction (89). At the same time, however, it has been suggested that CaMKII may mediate vasodilation in endothelial cells through its interaction with endothelial nitric oxide synthase (90, 91). In addition to its physiological roles in vascular biology, CaMKII also contributes to endothelial and smooth muscle dysfunction following injury. CaMKII promotes neointimal formation after carotid artery ligation and influences vascular smooth muscle cell migration, an event that has been attributed to ox-CaMKII-mediated increase in matrix metalloproteinase 9 (92–95). Ox-CaMKII has also been linked to vascular endothelial dysfunction from ischemia/reperfusion injury and aging (96, 97), further supporting the proposed role of ox-CaMKII in antagonistic pleiotropy (70).

### Metabolism

Calcium is a critical second messenger in cells for metabolic regulation, a relationship that may connect CaMKII to energy metabolism. ATP-generating enzymatic pathways are directly regulated by Ca^2+^ uptake by mitochondria. In cardiomyocytes, SR and mitochondria have direct, physical interactions that are important for regulation of metabolism and ATP production (98). CaMKII-dependent SR Ca^2+^ leak is linked to mitochondrial metabolic dysfunction, presumably due to changes in Ca^2+^ retention capacity of the mitochondria (99). Inhibition of extramitochondrial CaMKII protects cardiac mitochondria from Ca^2+^ overload, loss of inner membrane potential, increased ROS, and resulting arrhythmia in models of prediabetes or obesity (99, 100). CaMKII is also found in mitochondrial fractions (34) and has been shown to have direct effects on mitochondrial metabolism (33). Mitochondrial CaMKII enhances the activity of several enzymes in the TCA cycle, resulting in enhanced nicotinamide adenine dinucleotide production. Excessive activation of CaMKII in mitochondria ultimately leads to the loss of electron transport chain components and reduced ATP production (33). In contrast, selective inhibition of mitochondrial CaMKII protects hearts from ischemic injury (33, 34).

### Muscle Biology

Though less well-studied compared to cardiac muscle, CaMKII activity also increases following skeletal muscle contraction. Both exercise and direct neuronal stimulation of skeletal muscle are associated with an increase in autonomous CaMKII activity and accompanying T287 autophosphorylation (101, 102). This role of CaMKII activity in contraction may be related to targeting of the ryanodine receptor by CaMKII in skeletal muscle (103). Moreover, the ability of skeletal muscle to sustain tension following contraction also appears to be specifically ox-CaMKII mediated. Mice harboring a CaMKII mutation that renders it insensitive to ROS activation (MM/VV) showed greater muscle fatigue and worse performance during exercise (70). In addition to mediating contraction itself, CaMKII may also be important for contraction-induced glucose uptake by skeletal muscle (104). CaMKII orchestrates transcriptional regulation of genes in skeletal muscle via serum response factor, HDAC4, and myocyte-enhancer factor 2 (105, 106). Wang et al. (70) demonstrated that ox-CaMKII was responsible for a select group of skeletal muscle transcriptional changes. MM/VV mice showed a dampened transcriptional response to exercise compared to controls via RNAseq analysis. Finally, it was recently shown that CaMKII participates in muscle development and is activated in response to skeletal injury by promoting myoblast-to-myotube fusion, a critical event in both processes (107).

### Meiosis and Fertility

CaMKII has also been shown to play a regulatory role in cell cycle progression, most notably in the resumption of meiosis II following egg fertilization (108, 109). During fertilization, Ca^2+^ oscillations are induced by sperm-specific phospholipase Cζ (110). These Ca^2+^ spike signals are transduced in part through CaMKII, which helps drive meiotic exit through a pathway involving the degradation of cyclin B1 and securin (111, 112). CaMKIIγ is the predominantly expressed messenger RNA (mRNA) isoform in mouse oocytes. This isoform appears to be the one responsible for cell cycle regulation during fertilization, given that female mice in a genetic knockout model of CaMKIIγ were shown to be infertile due to egg activation defects (108). In addition to its role in meiosis, CaMKII may help regulate centrosome duplication, as inhibition of CaMKII was shown to prevent centrosome duplication in *Xenopus* extracts (113).

## CAMKII IN CARDIOVASCULAR DISEASES

### Heart Failure

Excessive CaMKII activation can contribute to cardiomyopathy and heart failure through a diverse set of processes. CaMKII has been implicated in the progression of pathological myocardial hypertrophy and dilated cardiomyopathy (114), maladaptive phosphorylation of Ca^2+^ handling proteins that contributes to arrhythmia, loss of myofilament contraction activating Ca^2+^ (115, 116), adverse metabolic reprogramming (33), activation of inflammation (38, 43, 46, 117), augmenting myocardial cell death (118, 119), and disrupting DNA repair (29). Studies examining cardiac tissue from control and heart failure patients have shown that cardiac CaMKII expression and activity are increased in failing human hearts (115) and in a variety of animal models of cardiac hypertrophy and heart failure. In failing human myocardium, CaMKII activity persists in spite of β-adrenergic receptor inhibition (120). Pressure overload hypertrophy induced by transverse aortic constriction (TAC) in murine hearts was accompanied by increased CaMKII expression and activity (114). Similarly, hypertrophied myocardium from spontaneously hypertensive rat models showed increased expression of CaMKIIδ (121). Interestingly, the changes seen in the spontaneously hypertensive rat model could be reversed by angiotensin converting enzyme inhibition, which also led to complete regression of myocardial hypertrophy in this model (122). Several genetic mouse models have confirmed a direct role for CaMKII in the development of pathological myocardial hypertrophy. Originally, transgenic mice overexpressing CaM developed severe cardiac hypertrophy (123), and this was subsequently shown to be associated with an increase in activated CaMKII (124). In contrast, mice with transgenic myocardial overexpression of AC3-I, a peptide inhibitor active against all CaMKII isoforms, are protected against a host of myocardial insults, including myocardial infarction (20, 125), calcineurin overexpression (126), inflammation (46, 117), infusion of isoproterenol (21, 125), angiotensin II (21), and aldosterone (20). Several additional lines of evidence parse discrete roles for splice variants of CaMKII in pathological cardiac remodeling. Transgenic mice that overexpress myocardial CaMKIIδ3/B, the splice variant that includes a nuclear localization signal, develop hypertrophy and dilated cardiomyopathy (124), but they are also protected from isoproterenol infusion, in part by augmenting CREB-mediated transcription of the mitochondrial Ca^2+^ uniporter (49). Mice with myocardial transgenic overexpression of CaMKIIδ2/C, a splice variant lacking a nuclear localization signal, developed profound myocardial hypertrophy, dysfunction, and premature death (114). Mice with targeted deletion of CaMKIIδ (knockout) showed attenuated hypertrophy after TAC (127) and an ameliorated heart failure phenotype (128), opening the door for CaMKII-based therapeutics for heart failure.

### Sudden Death and Arrhythmias

CaMKII has emerged as a nodal proarrhythmic signal in atrial fibrillation (129). Atrial fibrillation often coexists with heart failure (130) and sinus node dysfunction (131). In several animal and human studies of atrial fibrillation and heart failure, there is an increase in several CaMKII upstream signals such as ROS (27, 132, 133), renin-angiotensin-aldosterone system metabolites (134, 135), and catecholamines (136, 137) in atrial tissue or circulating plasma. Concomitantly, there is increased CaMKII expression and activation via phosphorylation and oxidation (129, 138) with consequent enhanced phosphorylation of various downstream targets such as RyR2 (116), PLN (139), and ion channels (140). RyR2 phosphorylation at S2814 increases opening probability of this intracellular Ca^2+^ channel (59), leading to increased SR Ca^2+^ leak into the cytoplasm, detected with fluorescent Ca^2+^ indicators and reported as intracellular Ca^2+^ sparks (141). SR Ca^2+^ release triggers delayed afterdepolarizations (27), which can trigger rapid atrial rhythms and atrial fibrillation. Targeted CaMKII inhibition, through either genetic manipulation or pharmacologic treatment, in various animal models, is protective against atrial fibrillation (27, 132, 142–144).

Similar to atrial fibrillation, CaMKII plays a key role in potentially lethal ventricular arrhythmias (ventricular tachycardia and ventricular fibrillation) in various forms of inherited and acquired cardiac diseases. CaMKII inhibition is a potential therapy in inherited diseases characterized by disordered intracellular Ca^2+^ handling such as RyR2-mediated catecholaminergic polymorphic ventricular tachycardia (CPVT) (145, 146), long QT syndrome (147, 148), Timothy syndrome with mutations in the L-type voltage-gated Ca^2+^ 1.2 channel (52), Barth syndrome (149), Duchenne muscular dystrophy (150), and ankyrin-B mutations (151). Acquired cardiac diseases associated with ventricular arrhythmias such as cardiac glycoside toxicity (152), alcoholic cardiomyopathy (153), heart failure, and myocardial hypertrophy appear to share a final common pathway: CaMKII-dependent RyR2 hyperphosphorylation with increased SR Ca^2+^ release (154–156), reduction of myofilament activator Ca^2+^, and proarrhythmia. Intriguingly, atrial fibrillation may contribute to heart failure by promoting CaMKII oxidation in the left ventricle (157). CaMKII inhibition in human tissue and in animal models of various genetic and acquired cardiac disease models has shown antiarrhythmic benefits.

### Myocardial Ischemia/Reperfusion and Infarction

Myocardial ischemia/reperfusion (I/R) and myocardial infarction involve a complex interplay of events and results in cardiac dysfunction. Excessive ROS, ox-CaMKII, and CaMKII activity are validated pathologic signals in myocardial infarction (21, 46) and I/R injury (45, 55). In addition, excessive intracellular and mitochondrial Ca^2+^ overload with consequent cardiomyocyte cell death via necroptosis and apoptosis through mitochondrial permeability transition pore opening, inflammatory signaling, and other CaMKII-dependent mechanisms are implicated in myocardial I/R injury and infarction (34, 38, 117, 118, 158, 159). CaMKII inhibition is protective against myocardial infarction (34, 46, 117, 119). Intriguingly, the effects of CaMKII on I/R injury appear to be dependent on the specific splice variant: While δ2/C and δ9 have both been shown to be detrimental, δ3/B overexpression protected mice from I/R injury (44). Thus, while total inhibition of cardiac CaMKII is protective, selective therapies that retain δ2/B activity hold the potential of even greater therapeutic benefit.

## CAMKII ANTAGONIST TOOLS AND CANDIDATE THERAPIES

**Figure 3:**
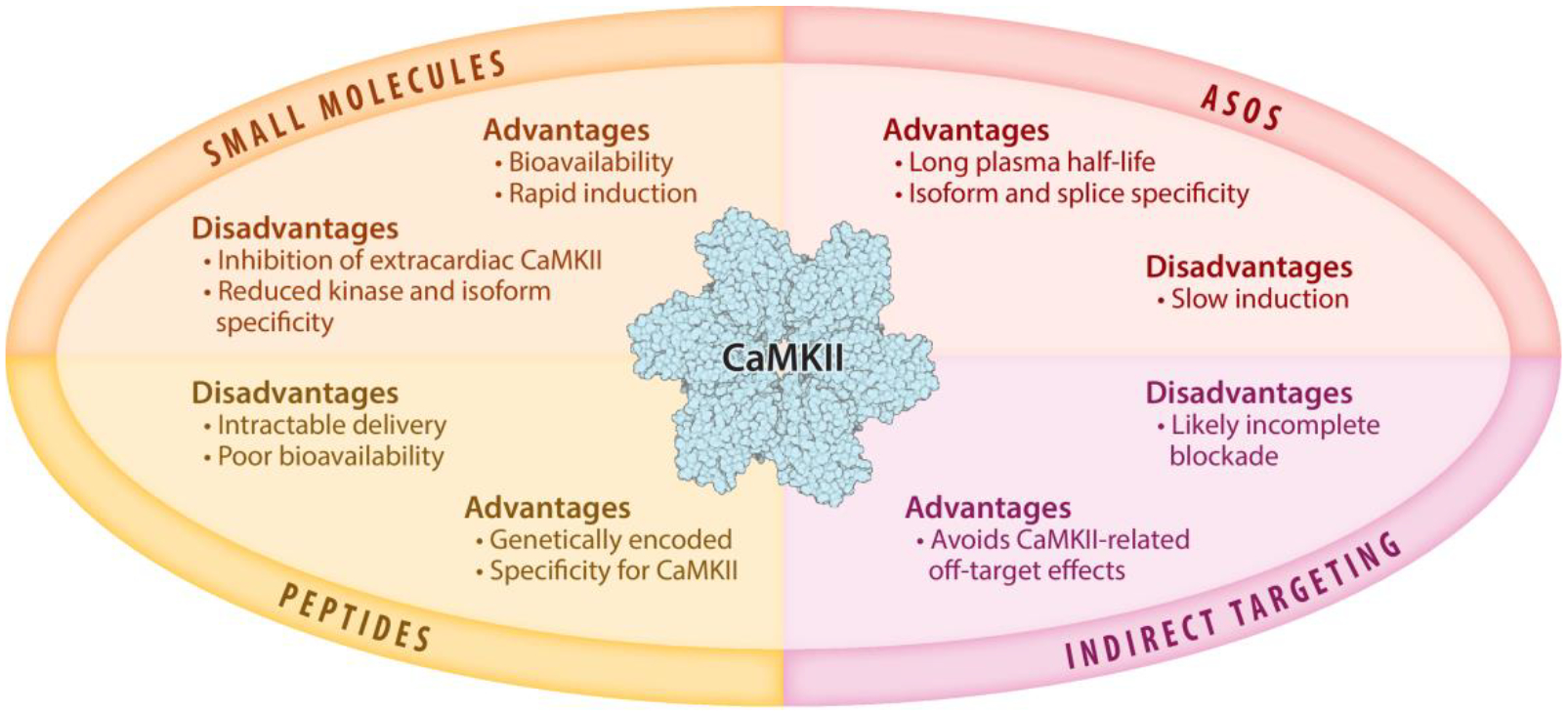
Advantages and disadvantages of various CaMKII targeting strategies. Abbreviations: ASO, antisense oligonucleotide; CaMKII, multifunctional Ca^2+^ and calmodulin-dependent protein kinase II. CaMKII structure from Protein Data Bank, rcsb.org (PDB ID 3SOA) (CC0 1.0), rendered with Biorender.com.

Given its established role as a driver of cardiac pathology, CaMKII inhibition has been an attractive therapeutic strategy for decades. Thus, a number of inhibition strategies have been developed, including small molecules, peptides, antisense oligonucleotides (ASOs), and targeting downstream CaMKII signaling. Due to various challenges, none of these approaches has yet led to clinical implementation. Here, we discuss progress and compare advantages and disadvantages of these approaches (Figure 3).

### Small Molecules

Small-molecule drugs have many attractive properties for cardiac targeting. They are usually highly bioavailable, are cell permeant, and have reasonable half-lives in plasma. Thus, they have dominated the cardio-pharmaceutical landscape for decades. Early efforts to develop CaMKII tool inhibitors yielded KN-93, which despite underappreciated shortcomings remains popular for preclinical research (160). Developed in 1991, this allosteric inhibitor prevents activation rather than inhibiting catalytic activity. KN-93 is troubled by its low potency (IC_50_ > 1 μM) and numerous off-target effects, including the L-type Ca^2+^ channel, Igr, and other kinases (161–165). This broad inhibitory profile not only confounds experimental results using this compound but precludes its use in humans. It is now believed that KN-93 is not a CaMKII inhibitor per se but rather a CaM antagonist (166, 167). This explains why KN-93 is ineffective against autophosphorylated (Ca^2+^/CaM independent) CaMKII activity and likely also explains the diverse range of off-target actions.

Recent efforts have yielded more potent and specific ATP-competitive small molecules. Allosteros Therapeutics developed AS105, a pyrimidine-based ATP competitor. AS105 has an in vitro IC_50_ of 8 nM and ameliorated Ca^2+^ dysregulation in adult cardiomyocytes from CaMKIIδC-overexpressing mice. In line with results from CaMKII knockout mice, CaMKII inhibition with AS105 does not affect baseline Ca^2+^ handling (168). Gilead Science’s GS-680 demonstrated impressive in vitro potency (IC_50_ of 2.3 nM) and was shown to selectively inhibit the CaMKIIδ isoform with a potency that was 3.1-, 8.7-, and 22.5-fold greater than for γ, α, and β, respectively (169). This selectivity was amplified in cellulo, where GS-680 had an EC_50_ that was 100-fold smaller in cardiomyocytes than in neurons. This would, in theory, lessen concerns for toxicity from inhibition of extracardiac CaMKII. In human cardiac tissue, GS-680 corrected proarrhythmic premature atrial contractions, SR Ca^2+^ leak, and afterdepolarizations. However, GS-680 caused decreased atrial systolic contractility. Sanofi’s RA306 was found to preferentially inhibit δ and γ isoforms with an IC_50_ of 15 and 25 nM, respectively (170). RA306 showed many desirable features, including low activity against hERG (IC_50_ >30 μM), oral bioavailability, and rescue of left ventricular ejection fraction in a genetic mouse model of dilated cardiomyopathy. A related compound, RA608, protected against pathological left ventricular remodeling after TAC surgery in mice when administered for 7 days and reduced SR Ca^2+^ leak in atrial myocardium from atrial fibrillation patients (171). Sumitomo Dainippon Pharma’s Rimacalib (SMP-114) was originally designed for rheumatoid arthritis and later repurposed as a CaMKII inhibitor (172). While less potent than KN-93 (IC_50_ 1–30 μM), Rimacalib was still able to ameliorate arrhythmogenic SR Ca^2+^ sparks in human and murine cardiomyocytes (173). Despite the promising features from these compounds, none have yet been translated into clinical use. Recently, NP202, a natural product and prodrug with moderate in vitro CaMKII inhibitory activity (IC_50_ of 250 nM) (174), failed to substantially affect left ventricular remodeling in patients presenting with myocardial infarction (175). However, interpretation of these apparently negative findings is complicated by lack of evidence that meaningful in vivo CaMKII inhibitory activity was achieved under the conditions of the clinical trial (176).

### Peptides

Peptide inhibitors are among the most potent CaMKII inhibitors. While they have served most of their history as research tools, advances in peptide delivery and modification could permit their use in humans. CaMKII inhibitory peptides can be categorized by their inspiration: CaMKIIN derivatives and pseudosubstrate mimetics. CaMKIIN derivatives started with the discovery of two endogenous genes that encode for CaMKII inhibitory peptides: CAMKIINα and CaMKIINβ (CAMK2N2/CAMK2N1) (87, 177). Both genes encode small peptides (78/79 amino acids) that inhibit CaMKII with an IC_50_ of ~50 nM and are expressed in the central nervous system. Notably, CaMKIIN expression is absent in the heart. These peptides have been subsequently optimized and truncated into minimal functional units to develop CaMKIINtide, CN21, and CN19o—the most potent CaMKII inhibitor to date (178). At just 19 amino acids, CN19o can inhibit CaMKII with an IC_50_ < 0.4 nM with little to no inhibition against CaMKI, CaMKIV, PKA, PKC, and AMPK. CN21 and CN19o have been leveraged into cell-permeable variants by fusion with the Tat peptide. TatCN19o has been shown to alleviate ischemic injury in the brain (179, 180). The second class of peptide inhibitors, substrate mimetics, are derived from the autoregulatory domain of CaMKII. By recreating the pseudosubstrate domain, but mutating T287 to phosphorylation-resistant alanine, these inhibitors sequester the catalytic domain. Because these peptides lack the CaM binding domain, they do not dissociate in the presence of Ca^2+^/CaM. These concepts led to the development of autocamtide-2-related inhibitory peptide (AIP) and AC3-I (181–183).

Peptide inhibitors offer several advantages over small molecules. So far, peptide inhibitors have proved more potent and selective than small-molecule inhibitors. They are genetically encoded, which could enable near-complete cardiac selectivity by using cardiac-biased viral vectors (such as AAV9) and cardiac-delimited promoters (such as cTnT). A recent study demonstrated this princi-ple by using AAV9 to deliver AIP to treat CPVT. AAV9 was able to selectively infect cardiac tissue, sustain robust expression, and ameliorate arrhythmogenesis, RyR2 Ca^2+^ leak, and CaMKII activity in CPVT mouse models and human induced pluripotent stem cell–derived cardiomyocytes (145). Furthermore, these peptides inhibit T287-autophosphorylated and MM281/282-oxidized CaMKII, the more hyperactive and harmful forms of CaMKII. Despite these advantages, peptide inhibitors face many obstacles for human use. Unmodified peptide delivery is intractable: They are not orally bioavailable, have half-lives in the order of minutes due to bloodstream proteases, require active and endocytic transport across cell membranes, and must survive intracellular proteases. While these obstacles have precluded use in humans, recent adjuvant technologies appear poised to address these concerns. A recent study developed inhalable nanoparticles that were able to deliver cardioprotective peptides to mouse hearts (184). Furthermore, peptide TANNylation has been shown to both protect peptides from plasma proteases and preferentially deliver them to myocardium. Delivery of cardioprotective basic fibroblast growth factor (bFGF) peptide via this method was able to reduce infarct size and improve cardiac function in mice (185).

### Nucleotides

In the past decade, ASOs have generated enthusiasm as a new class of therapeutics. While interfering RNA/DNA has been used as a research tool for decades, its use as a therapeutic was nonexistent due to toxicity, off-target effects, and short half-lives due to nucleases. However, recent improvements in nucleotide chemistry have yielded antisense molecules with half-lives in the order of weeks, cell type–specific permeability, and even oral bioavailability (186, 187). This has caused a boom in the development of ASO therapeutics, and several US Food and Drug Administration-approved ASO-based formulations are now available (188). ASOs function by base pairing with the mRNA target, thereby targeting it for degradation or hindering its expression. Careful design of a steric ASO can even bias mRNA splicing toward inclusion or deletion of exons. Thus, nucleic acid–based inhibition offers key advantages over both peptides and small molecules. Firstly, nucleotide base-pairing allows for genetic specificity at the isoform and splice variant selectivity; this would allow exclusive knockdown of the predominantly cardiac CaMKIIδ. More precise even, an ASO could permit downregulation of δC and δ9 splice variants while retaining the cardioprotective activity of δB. Pursuing these unique properties, Ionis Pharmaceuticals recently developed an ASO against CaMKIIδ. This led to a noticeable reduction in cardiac CaMKII and fewer arrhythmic events in mice that had undergone myocardial infarction (189).

### CRISPR

Having earned the 2020 Nobel Prize in chemistry, genome editing is poised to revolutionize many aspects of human life and disease. CRISPR is already being used in humans for the treatment of hematological disorders, and the effect is starting to spill over into the cardiovascular field. For example, animal proof-of-principle studies have corrected Duchenne muscular dystrophy and porphyria in cardiac cells (190, 191). Additionally, PSCK9 genome editing has already passed initial trials in nonhuman primates (192). While the most attractive application would be to directly knock out CaMKIIδ in cardiac tissue, data suggest that this would likely be infeasible in the near term for two reasons. First, use of CRISPR knockout in humans remains tenuous due to concerns of off-target nuclease activity and subsequent carcinogenesis. Secondly, mouse studies have shown that adult myocardium is quite resistant to Cas9 nuclease–mediated editing, with an efficiency of 15–45% (193). This incomplete knockdown may not be enough for a therapeutic benefit. Instead, nuclease-dead CRISPR technologies might offer more effective alternatives. CRISPR activation, whereby nuclease-dead Cas9 is fused to transcriptional activators and directed to a particular gene, could be used to specifically upregulate the CaMKII inhibitor CaMKIINα/β. This may be an elegant approach to leverage the body’s endogenous CaMKII inhibitor, which is natively not expressed in myocardium. Notably, it was recently shown that CRISPR activation is feasible and efficient in murine hearts (194).

### Indirect Targets

As an alternative to direct kinase inhibition, regulation of CaMKII PTMs, multimerization, and localization could be leveraged to quell hyperactivity. As previously mentioned, myriad proteins can modify CaMKII: Phosphatases such as PTENα, PP1, PP2A, and PP2C have been implicated in dephosphorylating T287 and T306/307 (195–197); receptor interacting protein 3 (RIP3) was found to phosphorylate and activate CaMKII at T287 (118); MICAL1 and its stereospecific reductase partner MsRB regulate the redox status of inhibitory M308; and *S*-stereospecific reductase MSRA leads to decreased oxidation at MM281/282 (20). Modulating these enzymes could shift the balance from activating to inhibiting PTMs. Work from our group found a mutant MICAL1 that shows selective loss of actin targeting and retains protection against CaMKII hyperactivation, a property that therapeutics could emulate (22).

The unique association domain–dependent oligomerization of CaMKII distinguishes it from other kinases. Conceivably, drugs that weaken the association domain interactions can indirectly affect the Ca^2+^ sensitivity, memory property, and subunit exchange of CaMKII. Indeed, it was recently reported that a neuroprotective brain metabolite, γ-hydroxybutyrate, acts by directly interacting with and stabilizing the association domain complex of CaMKIIα (198). Additionally, the discovery of CaM-KAP, which recruits CaMKII to the SR, opens the possibility to redirect CaMKII localization without inhibition (32). These approaches, however, are not without challenges. For one, inhibition via a single PTM or indirect target may not be enough to offset other activating modifications. Phosphatase modulation also suffers from lack of selectivity, since phosphatases are highly promiscuous and act on many more targets, on average, than kinases. Lastly, prevention of oligomerization and redirection away from T-tubules may not prevent deleterious effects on DNA damage, contractile function, and inflammation.

## CONCLUSIONS

Decades of research have revealed CaMKII to be a fascinating and nuanced physiological signal and an indisputable agent of cardiovascular and other diseases. It appears the field is at an exciting juncture, armed with new therapeutics capable of unprecedented selectivity. These tools may provide the long-sought-after therapeutic implementation of CaMKII inhibition but will likely require new information to understand how individual CaMKII isoforms and splice variants operate in health and disease.
